# Unique Case of Congenital Lipomatous Overgrowth With Vascular Malformations, Epidermal Nevi, and Skeletal/Spinal Anomalies Syndrome in a Pediatric Patient

**DOI:** 10.7759/cureus.10737

**Published:** 2020-09-30

**Authors:** Kirby E Quinn, Juan Infante, Willa Thorson, Chad M Thorson

**Affiliations:** 1 Dewitt-Daughtry Family Department of Surgery, Division of Pediatric Surgery, University of Miami Miller School of Medicine, Miami, USA; 2 Department of Radiology, University of Miami Miller School of Medicine, Miami, USA; 3 Department of Human Genetics, University of Miami Miller School of Medicine, Miami, USA

**Keywords:** vascular malformations, cloves syndrome, lipomatous, pediatric

## Abstract

Vascular malformations are being increasingly identified with associated syndromes caused by sporadically occurring, non-heritable somatic mutations. CLOVES syndrome is a newly recognized constellation of congenital lipomatous overgrowth with vascular malformations, epidermal nevi, and skeletal/spinal anomalies. We report the unique case of CLOVES syndrome diagnosed in a pediatric patient five years after the initial surgical resection of an extensive venolymphatic malformation involving the chest, neck, axilla, and posterior trunk. The lipomatous overgrowths were successfully resected, and subsequent genetic analysis revealed a heterozygous, pathogenic, somatic variant in the PIK3CA gene, confirming our suspicion of CLOVES syndrome.

## Introduction

Vascular malformations are being increasingly identified with sporadically occurring non-heritable somatic mutations. Congenital lipomatous overgrowth with vascular malformations and epidermal nevi was first coined in 2007 [1]; then in 2008 it was amended to reflect the spinal manifestations of the syndrome [2] and has been referred to as congenital lipomatous overgrowth with vascular malformations, epidermal nevi, and skeletal/spinal anomalies (CLOVES) ever since. It is thought to be due to a somatic activating mutation in PIK3CA during early embryonic development [3-9]. CLOVES syndrome has clinical overlap with other syndromes and can be misdiagnosed as Kippel-Treanuay syndrome (KTS), Proteus syndrome (PS), or hemi-hyperplasia lipomatosis syndrome (HHML) [1,3,8,10,11]. Truncal overgrowth and characteristic macrodactyly differentiate CLOVES from other overgrowth syndromes [11,12]. It is important to distinguish CLOVES syndrome from other overgrowth conditions due to differential disease courses, management, and prognosis. However, optimal management is difficult secondary to the heterogenous nature and rarity of the condition, as less than 200 cases have been documented to date [13].

Although there has been reported success with the use of oral rapamycin, which inhibits the mammalian target of rapamycin (mTOR) pathway that is activated in CLOVES syndrome [5,14], data is still lacking, and the lipomatous overgrowths and vascular malformations often require surgical intervention [4,11,15]. However, surgical intervention can be challenging owing to the invasive nature of these overgrowths and possibility of recurrence and disfiguration [14]. We report our own unique case of confirmed CLOVES syndrome with the identification of a phosphatidylinositol-4,5-bisphosphate 3-kinase catalytic subunit alpha (PIK3CA) pathogenic allele that was diagnosed five years after the initial surgical resection of an extensive venolymphatic malformation and subsequent successful lipomatous overgrowth resections. 

## Case presentation

**Figure 1:**
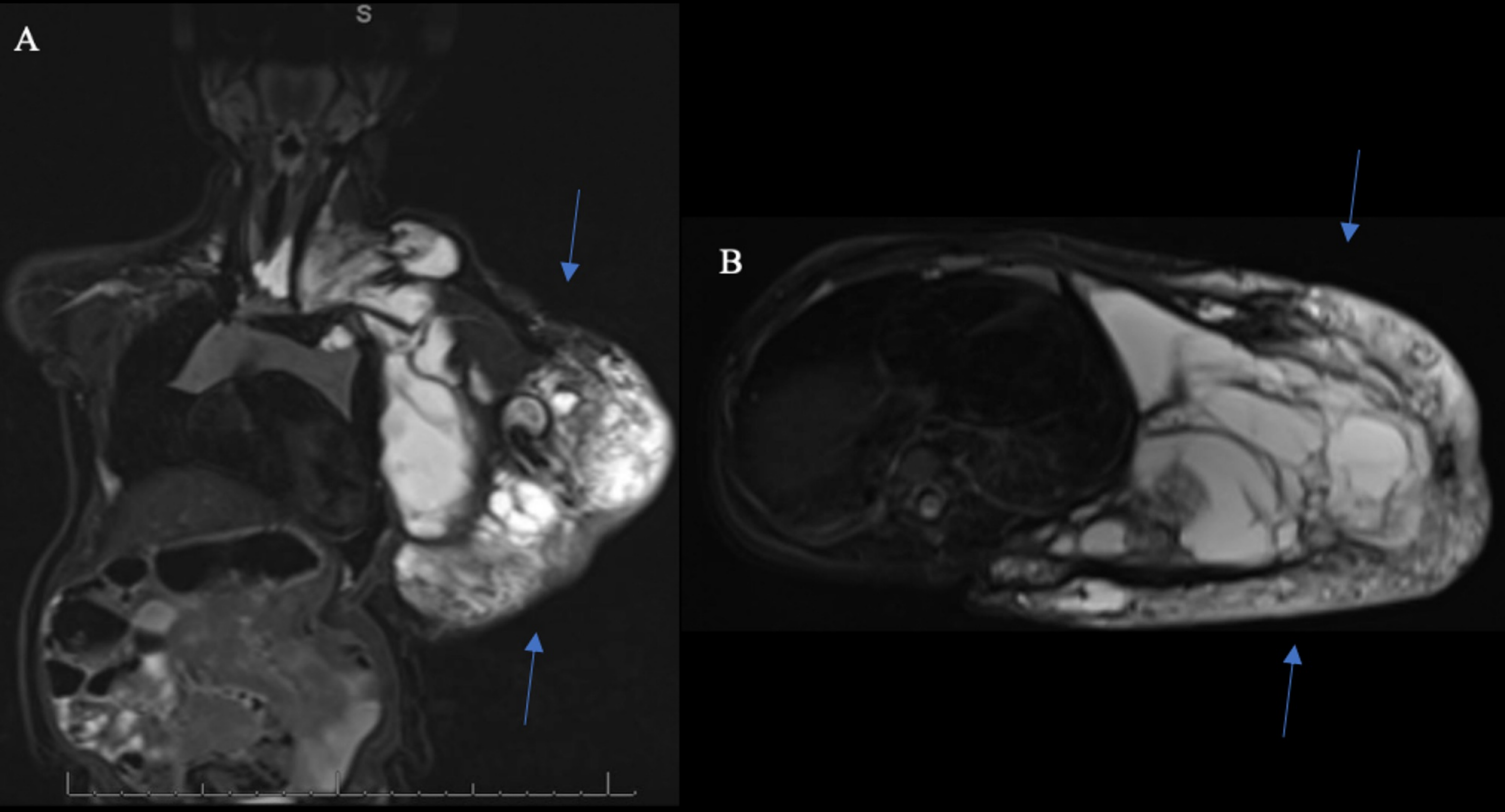
Imaging of Venolymphatic Malformation T2-weighted spin-echo images with fat-saturation in the coronal (A) and axial (B) planes show a large, spectated, multispatial lesion with fluid-fluid levels consistent with a venolymphatic malformation. The lesion extends from the left anterior chest to the back across the supraclavicular and suprascapular regions. The left lateral chest wall including the axillary regions contain a large bulk of the lesion.

We report the unique case of a six-year-old female treated for an extensive venolymphatic malformation noted at birth. The lesion was centered around the left axilla as well as chest wall and also involved significant areas of the neck and posterior trunk (Figure 1A, 1B). The patient also had lesions on bilateral posterior scapular regions and the left breast. At the age of two, she underwent combined surgery with pediatric and plastic surgery where the axillary/chest wall lesion was resected along with significant neurolysis off the brachial plexus and flap closure.

Surgical pathology of the mass revealed a 25.5 cm x 17.5 cm x 7.0 cm combined venous and lymphatic malformation with areas of cystic formation, myxoid change, and focal sclerosis. The pediatric surgeon at our institution deemed the debulking surgery of the lymphangioma to be successful and recommended waiting until the age of five to resect the residual neck and back disease. This was advised due to the encasement of neurovascular structures within the residual malformation, including the brachial plexus. Additional recommendations included postponing resection of the left breast lesion until breast development was complete, to avoid disrupting the developing breast bud.

**Figure 2:**
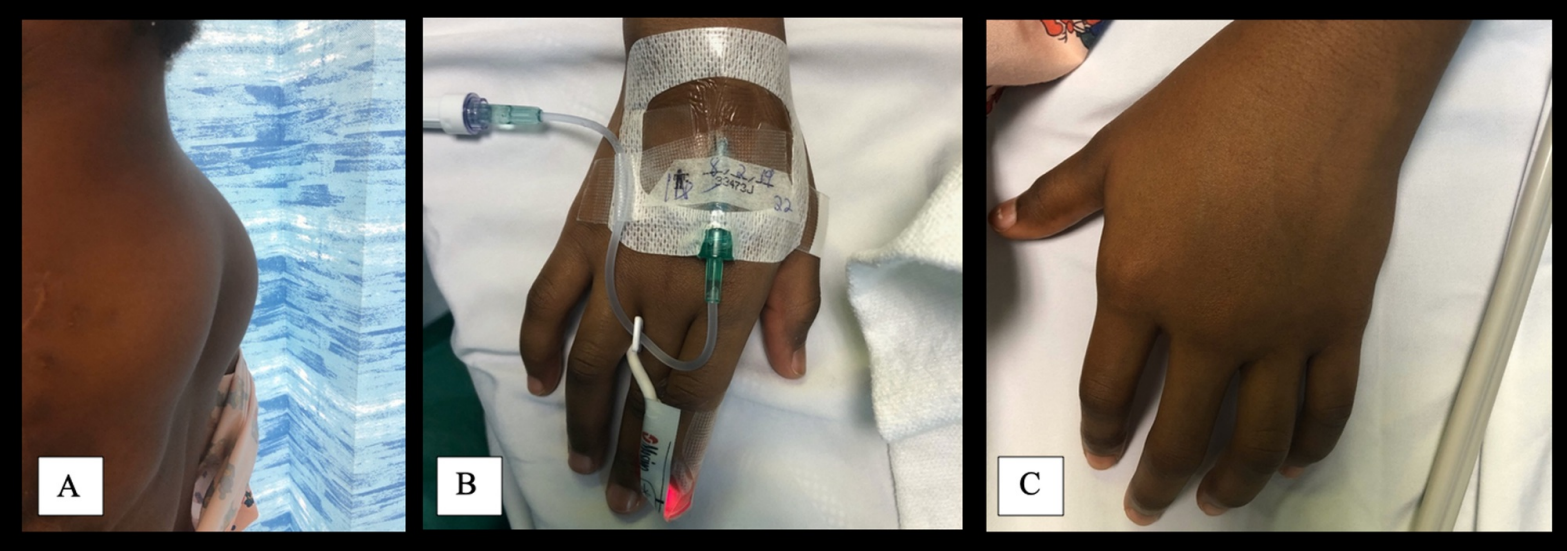
Hand Asymmetry and Lipomatous Overgrowths Large lipomatous overgrowths found on presentation five years after resection of the left axillary/chest wall venolymphatic malformation (A). Hand asymmetry noted between smaller normal-sized right-upper extremity (B) compared to enlarged left-upper extremity (C).

The patient presented again to our institution at the age of six for follow-up. Physical exam revealed bilateral, mobile, subcutaneous lesions in the scapular region raising suspicion for large lipomas (Figure 2A). In addition, asymmetry of the upper extremities was noted in the left forearm (Figure 2C) and hand twice as large as that in the right forearm (Figure 2B). Given the constellation of symptoms, a diagnosis of CLOVES was suspected. Preoperative magnetic resonance angiogram (MRA) was obtained of the neck, chest, and abdomen. MRA revealed multiple small foci of microcytic lymphatic malformations in the chest at the site of prior resection with irregularity of the left subclavian vein with absence of the left axillary vein and multiple collateral vessels in this area concerning for thrombosis. The patient underwent successful excision of the lipomatous overgrowths. Pathology demonstrated a 9.0 cm x 6.0 cm left and 7.0 cm x 4.0 cm right lipomatous overgrowth. Outpatient genetics consultation was scheduled.

Genetic consultation revealed that the patient had a history of developmental delay and did not walk until 18 months of age or talk until age three. However, this has not significantly impacted the patient’s current school performance. There is no family history of significant genetic illness, consanguinity, lipomatous overgrowths, macrocephaly, or significant nevi. Physical examination showed appreciable immobility of the left-upper extremity and neck asymmetry with the left shoulder inferior to the right with bilateral shoulder sloping. Skin examination showed a left-sided breast mass, soft and mobile to palpation, with skin hyperpigmentation, one café-au-lait spot on the right flank, and raised areas of hyperpigmentation over the left breast mass, and surrounding scar of the previously resected lymphangioma. Her head circumference was normal for her age, and no nevi were noted aside from the café-au-lait spot.

Definitive diagnosis of CLOVES syndrome was made after genetic testing revealed a heterozygous, pathogenic, missense variant in the PIK3CA gene, with positive testing of the affected tissue and negative testing of her peripheral blood. The patient also underwent a postoperative cardiac workup, which was negative for any structural abnormalities of the heart.

## Discussion

In general, syndromes consisting of complex vascular malformations and overgrowth are rare, underrecognized, and pose a diagnostic and management challenge [12]. When a patient presents with a mixed or complex type of vascular malformation, associated anomalies such as lipomatous overgrowths or skeletal malformations should raise clinical suspicion for CLOVES syndrome, rather than an isolated congenital vascular malformation. Patients with this constellation of symptoms should be referred for genetic testing to confirm the diagnosis. Prior to genetic testing, the differential diagnoses for this patient and her presentation included KTS, PS, HHML, Bannayan-Riley-Ruvalcaba syndrome (BRRS), and CLOVES syndrome.

KTS can cause mixed vascular malformation as seen in CLOVES; however, it typically affects the lower extremities, with sparse truncal involvement [11,12]. Features of BRRS include macrocephaly, developmental delay, and lipomatosis [12] but was low on the differential because it typically does not have truncal lipomatosis, which is a key characteristic in CLOVES syndrome [11]. PS has a postnatal onset of severely deforming but localized skeletal overgrowth with poor prognosis [1,10]. Our patient did not appear to have progressive growth of the malformations or skeletal anomalies during the time from the initial lymphangioma resection to when she presented for resection of the lipomatous overgrowths. Therefore, the lack of progressive nature of our patient’s presentation made PS less likely. HHML was high on the list for this patient, but HHML is more characterized by capillary malformations [1,12], typically lacking the deeper mixed vascular malformation as evident in our patient. Overall, CLOVES syndrome was most likely due to the mixed truncal vascular malformation, lipomatous overgrowths, and upper extremity skeletal anomalies present in our patient.

Ultimately, CLOVES syndrome was confirmed in our patient by genetic testing of her blood and affected tissue. No mutations were identified in her blood, but testing of her affected tissue revealed a heterozygous, pathogenic, missense variant in the PIK3CA gene (p.E545K). Given the presence of the pathogenic variant in her tissue and not her blood, this confirmed the somatic nature of her variant, which is consistent with what has been previously reported in CLOVES syndrome [7].

The vascular malformations in these patients may be complicated, invasive, and include mixed components of capillary, lymphatic, venous, and arteriovenous malformations [12]. Additionally, overgrowths typically involve adipose tissue, but all tissue types can be involved [3]. This poses management difficulties with regard to surgical resection. There have been mixed results with attempts at lipomatous resection and instances of recurrence [11]. In our case, the surgeon purposefully left behind residual overgrowth during the initial venolymphatic surgical resection due to neurovascular encasement and concerns interfering with the patient’s future breast development. Surgical management of these patients will often require a multidisciplinary approach with consideration to not cause further disfiguration. Additionally, there is a chance of recurrence due to the somatic activating mutation involved in the pathogenesis of the overgrowths. 

In 2012, a search for somatic mosaic mutations from six affected individuals revealed a missense PIK3CA mutation in the affected tissue specimens [6]. A unique and challenging aspect of managing this rare syndrome is the different and non-overlapping phenotypes that may manifest. This spectrum of phenotype differences may be explained by the precise time during embryonic development that the somatic mutation developed, and depending on the stage of embryonic development the mutation occurred, the phenotype of the syndrome may differ among patients [6]. Since this syndrome has a heterogenous presentation and diagnosis is difficult, genetic testing and analysis is of the utmost importance upon clinical suspicion of CLOVES syndrome. Genetic confirmation of a PIK3CA somatic variant may allow clinicians to learn more about the manifestations of CLOVES to better standardize treatment and optimize surgical resection of the vascular malformations and overgrowths in these patients.

## Conclusions

CLOVES syndrome is a rare and newly delineated syndrome of a non-inherited somatic mutation resulting in congenital lipomatous overgrowths, vascular malformations, epidermal nevi, and spinal/skeletal anomalies. There is still a limited amount of literature regarding management of the syndrome; thus, clinicians suspecting CLOVES syndrome should refer for genetic testing to confirm a PIK3CA somatic variant, to allow for further research into this rare condition. Surgical resection of overgrowths in CLOVES syndrome can be complicated and often requires multiple surgical procedures; therefore, we recommend a multidisciplinary approach to surgical planning and management. Surgical resection should be aimed to preserve surrounding structures and to avoid disfiguration. We report the unique case of a successful staged resections of venolymphatic and lipomatous overgrowths and identification of a PIK3CA pathogenic allele leading to diagnosis of CLOVES syndrome.
